# Induction of epstein-barr virus (EBV) lytic cycle in vitro causes lipid peroxidation, protein oxidation and DNA damage in lymphoblastoid B cell lines

**DOI:** 10.1186/1476-511X-10-111

**Published:** 2011-07-01

**Authors:** Bochra Gargouri, Rihab Nasr, Malek Mseddi, Riadh benmansour, saloua Lassoued

**Affiliations:** 1 Unité de Biotechnologie et Pathologies, Institut Supérieur de Biotechnologie de Sfax, Tunisia

**Keywords:** B95-8 cell line, Raji cell line, oxidative stress, Epstein Barr Virus, lymphoblastoid cell line

## Abstract

**Background:**

We investigated the oxidative modifications of lipids, proteins and DNA, potential molecular targets of oxidative stress, in two lymphoblastoid cell lines: B95-8 and Raji, after EBV lytic cycle induction. Conjugated dienes level was measured as biomarker of lipid peroxidation. Malondialdehyde adduct and protein carbonyl levels, as well as protein thiol levels were measured as biomarkers of protein oxidation. DNA fragmentation was evaluated as biomarker of DNA oxidation.

**Results:**

After 48 h (peak of lytic cycle), a significant increase in conjugated dienes level was observed in B95-8 and Raji cell lines (p = 0.0001 and p = 0.019 respectively). Malondialdehyde adduct, protein carbonyl levels were increased in B95-8 and Raji cell lines after EBV lytic cycle induction as compared to controls (MDA-adduct: p = 0.008 and p = 0.006 respectively; Carbonyl: p = 0.003 and p = 0.0039 respectively). Proteins thiol levels were decreased by induction in B95-8 and Raji cell lines (p = 0.046; p = 0.002 respectively). DNA fragmentation was also detected in B95-8 and Raji cell lines after EBV lytic cycle induction as compared to controls.

**Conclusion:**

The results of this study demonstrate the presence of increased combined oxidative modifications in lipids, proteins in B95-8 and Raji cells lines after EBV lytic cycle induction. These results suggest that lipid peroxidation, protein oxidation and DNA fragmentation are generally induced during EBV lytic cycle induction and probably contribute to the cytopathic effect of EBV.

## Background

Epstein Barr virus (EBV) is a ubiquitous gammaherpesvirus that infects more than 90% of the human population. The EBV can infect its target cells in both a latent and a lytic mode. The latent cycle is characterized by the expression of six Epstein-Barr nuclear antigens (EBNAs), three latent membrane proteins LMP1, LMP2A, and LMP2B, two untranslated RNAs (EBER), and a family of transcripts from the BMH1A region of the genome [1,2]. Upon EBV reactivation, two key immediate early (IE) lytic genes, BZLF1 and BRLF1, encoding Zta (BZLF1 transcription activator) and Rta (BRLF1 transcription activator) respectively, are transcribed, and consequently activate several downstream viral promoters and lead to an ordered cascade of viral gene expression [3]. The latent form of EBV can be induced to enter the lytic form in vitro by treatment with various chemicals, including 12- 0-tetradecanoylphorbol-13-acetate (TPA) [4], halogenated pyrimidine [5], n-butyrate [6], calcium ionophores [7]. These inducers act through different signalling pathways to transactivate gene of EBV, which encodes the ZEBRA protein. The expression of ZEBRA protein then transactivates immediate early and early genes of EBV, and thereby induces the lytic cycle [8,9].

EBV is associated with a spectrum of malignancies of lymphoid and epithelial cell origin, such as Burkitt's lymphoma [10], nasopharyngeal carcinoma (NPC) [11], and gastric carcinoma [12]. The role of EBV in malignancy was largely studied and latent EBV antigens such as LMP or EBNA were generally incriminated [13-15]. The fact that all EBV-associated malignancies have a predominantly latent pattern of viral gene expression led to the assumption that only the latent phase of viral gene expression is important during the development of EBV associated malignancies. Nevertheless, a small number of lytically infected cells are frequently detected in biopsies of EBV-associated lymphoproliferative diseases (LPDs) in immunosuppressed individuals. Lytic replication has been observed at the site of tumor development in posttransplant lymphoproliferative disorder (PTLD) [16] as well as in Burkitt's lymphoma [17]. In addition, Hong et al. have shown that EBV mutants that cannot undergo lytic viral replication are defective in promoting EBV-mediated LPD [18]. Moreover, an increasing number of diseases were found to be associated with EBV lytic cycle and exhibit an oxidative stress state at the same time, such as rheumatoid arthritis [19,20], and infectious mononucleosis [21].

A lot of evidences demonstrated that EBV is implicated in the genesis of oxidative damages in vitro or in vivo and that could participate in the pathogenicity of the EBV. An excessive production of reactive oxygen specie (ROS) was highlighted in EBV positive lymphoblastoid cell lines transformed in vitro [22] or derived from Burkitt's lymphoma [23]. In addition, oxidative damage was determined during the course of acute EBV infection [24] and replication [25] in lymphoblastoid cell lines.

Oxygen free radicals or, more generally, ROS, are products of normal cellular metabolism. ROS are well recognised for playing a dual role as both deleterious and beneficial species, since they can be either harmful or beneficial to living systems [26]. Damage has been reported to occur on all components of biological systems (DNA, RNA, lipids, proteins, carbohydrates, low-molecular-mass species, antioxidants) due to the high reactivity of many oxidants [27]. Proteins are likely to be major targets, as a result of their abundance in cells (proteins compose 70% of the drymass of most cells), and their rapid rates of reaction both with many radicals and with other oxidants (peroxides, excited states, peroxynitrite, chloramines, ozone) [27]. Otherwise, DNA damage is one of the more reliable markers to detect oxidative stress [28,29].

However, there is no adequate knowledge in the literature about protein and DNA modifications in cells after EBV lytic cycle induction. Many different types of protein oxidative modifications can be induced by free radicals [30,31]. Protein carbonyl, protein thiol formation had been accepted as a phenomenon of protein oxidation [32].

This study sought the effect of virus replication on lipids, proteins and DNA oxidation of two lymphoblastoid cells lines B95-8 and Raji. The induction of the lytic cycle was done by TPA. The lipids, proteins and DNA damages were then determined after 48 h, the peak of lytic cycle [33], by measuring the level of conjugated dienes as parameters of lipids peroxidation, malondialdehyde adduct, protein carbonyl and protein thiol as parameters of proteins oxidation, and the DNA fragmentation assays as marker of DNA damage.

## Results

### Evaluation of lipid peroxidation (LPO)

**Figure 1:**
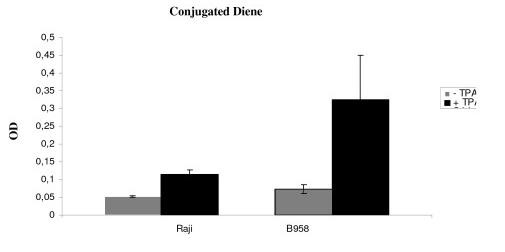
I**llustrates conjugated diene level**. To evaluate lipid peroxidation, CD level was measured 48 h after induction of the lytic cycle. The study was performed in parallel cultures with or without TPA treatment. After 48 h, a significant increase in CD level was observed in B95-8 and Raji cell lines (p = 0.019 and p = 0.0001 respectively).

CD level was measured 48 h after induction of the lytic cycle to evaluate lipid peroxidation. The study was performed in parallel cultures with or without TPA treatment. Our data show a significant rise in CD level in B958 and Raji cell lines after EBV lytic cycle induction (p = 0.0001 and p = 0.019 respectively) (Figure 1).

A comparison of differences between basal and post induction CD level was monitored. A significant rise in CD level was observed in B958 cell line as compared to CD level in Raji cell line (p = 0.01).

### Evaluation of MDA adduct

**Figure 2:**
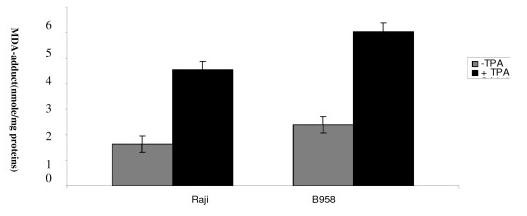
**Illustrates MDA adduct level**. MDA adduct level was measured in B958 and Raji cell line after 48 h of lytic cycle induction. Malondialdehyde adduct level was increased in B95-8 and Raji cell lines after EBV lytic cycle induction as compared to controls (p = 0.008 and p = 0.006 respectively).

MDA adduct level was monitored in B958 and Raji cell lines after 48 h of lytic cycle induction. MDA adduct level was low in B958 and Raji cell line without TPA treatment. Our data show a significant rise in MDA adduct level in B958 and Raji cell lines after EBV lytic cycle induction (p = 0.008 and p = 0.006) (Figure 2).

The deviation between basal level and post induction level of MDA adduct was higher in B958 than in Raji cell line. (p = 0.08).

### Evaluation of protein carbonyl and SH level

**Figure 3:**
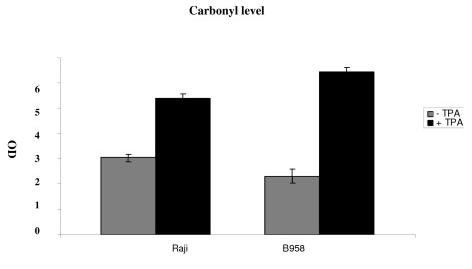
**Illustrates protein carbonyl level**. PC level was assessed in B958 and Raji cell line after 48 h of lytic cycle induction to determine protein oxidation. A significant rise in PC level was observed in B958 and Raji cell line 48 h after EBV lytic cycle induction as compared to controls (p = 0.0030 and p = 0.0039 respectively).

**Figure 4:**
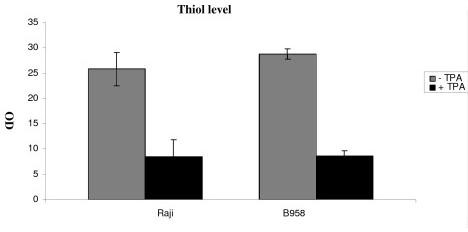
**Illustrates protein thiol level**. Proteins thiol was monitored in B958 and Raji cell lines after 48 h of lytic cycle induction. Proteins thiol level was decreased by induction in B95-8 and Raji cell lines (p = 0.046; p = 0.002 respectively).

PC and SH levels were assessed in B958 and Raji cells after 48 h of lytic cycle induction to determine proteins oxidation. A significant rise in PC level was observed in B958 and Raji cell lines, 48 h after EBV lytic cycle induction as compared to controls (p = 0.0030 and p = 0.0039 respectively). Protein thiol level was decreased by induction in B95-8 and Raji cell lines (p = 0.046; p = 0.002 respectively) (Figure 3 and 4).

As evidenced with CD and MDA adduct, our result show a significant rise in carbonyl level as well as a significant decrease in SH level in B95-8 than in Raji cell line (p = 0.01 and p = 0.01 respectively).

### Evaluation of DNA fragmentation

**Figure 5:**
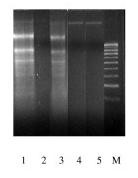
**Agarose gel analysis of DNA from B958 and Raji cells**. The study was performed in parallel cultures with or without TPA treatment. TPA treatment (lanes 1 (B958) and 3 (Raji)), untreated cells (lanes 2 (B958) and 4 (Raji)) and untreated lymphocytes used as control (lane 5). B95-8 and Raji cells were harvested at 48 h after EBV lytic cycle induction, and analyzed by 2% agarose gel. EBV lytic cycle induction causes DNA fragmentation in B95-8 as well as Raji cells.

To investigate the DNA fragmentation, B95-8 and Raji cells were treated with 8 nM TPA, and DNA was isolated and analyzed by agarose gel electrophoresis. These experiments demonstrated that DNA from B95-8 and Raji cells after EBV lytic cycle induction was fragmented (Figure 5).

## Discussion

EBV is implicated in the generation of oxidative stress both in vivo, in some EBV associated malignancies and diseases such as NPC [34] and in vitro, in EBV-infected cell lines [23].

In a previous report, we demonstrated that EBV lytic cycle induction in lymphoblastoid cell line is responsible for the occurrence of oxidative stress by an increase in MDA level and a disturbance of SOD and CAT activities [25]. However, the direct role of EBV replication or the expression of lytic viral antigens in the genesis of proteins and DNA oxidation has not been investigated.

For these reasons, we investigated the effect of lytic cycle induction on the proteins oxidation, lipids peroxidation and DNA fragmentation of two lymphoblastoid cells lines: B958 and Raji. We chose to work with the minimal and sufficient concentration of TPA inducing EBV lytic cycle which is 8 nM. This concentration is very low, compared to the concentrations that will induce oxidative stress (1.5-15 μM) [22], and this dose did not trigger stress in the EBV negative cell line (DG75) treated in the same conditions [35].

A significant rise in CD levels was observed in Raji and B958 cell lines 48 h after EBV lytic cycle induction. We observed an increase in CD level in the two cell lines, indicating the presence of oxidative stress as a consequence of induction of the lytic cycle.

Many different types of protein oxidative modifications can be induced by free radicals. Their oxidative damage can be evaluated by different ways, such as protein carbonyls, MDA adduct as well as SH level.

Protein carbonyls determination has been particularly proposed as a marker of oxidative stress, since they form early and they are generally stable [32].

Our data have show a significant rise in PC and MDA adduct levels as well as a decrease in SH level in Raji and B958 cells line, 48 h after EBV lytic cycle induction. Taken together, these results demonstrate protein oxidation during lytic cycle induction. Oxidized proteins cause major physiological perturbations including loss of structure and function [36]. Besides, previous studies have show that oxidatively modified proteins lead to the genesis of new epitopes that incite autoantibody production. Protein oxidation causes conformational changes, which include the exposure of the hydrophobic regions of the proteins, explaining the increased interaction between antibodies and antigens after oxidation [36]. In fact, an increasing number of autoimmune diseases were found to be associated with EBV lytic cycle and exhibit an oxidative stress state at the same time, such as rheumatoid arthritis [36].

Our data demonstrated that oxidative damage was higher in B958 as compared to Raji cell line. Interesting, B958 cell line can produce active EBV viral particles [37], contrarily to Raji cell line. So, our results reinforce our suggestion in our previous study, in which we suggested that an EBV viral program further down the lytic pathway may be responsible for observed oxidative stress. Raji is a Burkitt's lymphoma-derived cell line, which does not produce viral particles, because of the total inhibition of viral DNA synthesis [4,38]. However, lipid peroxidation and protein oxidation were observed after EBV lytic cycle induction, indicating that virus replication is not required for inducing oxidative stress. Other viral antigens, which continue to be expressed in Raji such as BZLF1, BRLF1, DNA polymerase, TK, and DNase, were probably responsible for the observed oxidative damage [39-41].

DNA fragmentation was detected in B95-8 and Raji cell lines after EBV lytic cycle induction as compared to controls. In fact, DNA damage is described in biopsies nasopharyngeal carcinoma patients [42], where EBV seems to play a crucial role [43]. Our results are in harmony with Michiko study, who reported a fragmentation of chromosomal DNA during EBV lytic cycle induction in Raji cell line [44]. Michiko suggested that EBV-specific early proteins participate in fragmentation of chromosomal DNA, since Phosphonoacetic acid, an inhibitor of EBV DNA polymerase, did not inhibit fragmentation of chromosomal DNA.

In our study, oxidative stress causes lipid peroxidation and protein oxidation. In addition, it is well known that ROS induce DNA strand breaks in human lymphocytes [45]. For these reasons, we suggest that ROS production causes DNA fragmentation. Our results is not in contradiction to thus presented by Michiko, since it has been shown that ROS regulate a spectrum of cellular responses, including P53, heat shock proteins, transcription factor NF-κB, and AP-1 [46-51]. Among them, P53, NF-κB, and AP-1 have been reported to regulate Epstein-Barr virus immediate-early gene BZLF-1, the key molecule responsible for the reactivation of EBV [49-51]. Also, we have previously reported that H_2_0_2 _and FeSO_4 _induce BZLF-1 gene expression [24]. However, whether ROS productions, during lytic cycle induction induce DNA degradation directly or indirectly by other protein expression has to be defined.

It is well know that cytokines were among those first reported to generate ROS in nonphagocytic cells. It is generally accepted that ROS generated by these ligand/receptor-initiated pathways can function as true second messengers and mediate important cellular functions such as proliferation and programmed cell death [52]. However, in lymphoblastoid cell lines, EBV lytic cycle induction downregulate cytokine genes expression such as (TNF)-α and interferon (INF)-γ receptor [53,54], and inhibits interferon regulatory factor (IRF)-7 mediated induction of IFN-α and β genes expression [55].

In lymphoblastoid cell line, several pathways could be a source of ROS production such as mitochondria or NADPH oxydase. In fact, Lajeunesse et al reported that BZLF-1 gene expression in human cell lines results in a dramatic reorganization of mitochondria accompanied by a significant alteration of mitochondrial membrane potential, which can be a sign of an increase in ROS production caused by the damage of the mitochondrial electron transport system [56].

Martin et al. reported that infection of neutrophils by EBV in vitro, rapidly reduces their survival, as confirmed by DNA staining with propiduim iodide and Hoechst 33342 and by DNA analysis [57]. Larochelle et al. demonstrated that in vitro EBV infection drastically increases the rate of spontaneous neutrophils apoptosis [58]. Flow cytometry analyses have revealed that 77% of neutrophils were apoptotic after 20 h post-infection, as compared to 22% in uninfected cells. However, the mechanism initiating the apoptotic process in EBV infected neutrophils remains unknown. Valko et al. reported an inhibition of protein kinase C (PKC) translocation and activation during EBV lytic cycle. It is well know that PKC was described as a source of ROS production [26].

In lyphoblastoid cell line, we described an oxidative stress status, 48 h after EBV activation, which correspond of a peak of EBV lytic cycle induction and we don't observe death of cells.

Hitochi et al. reported that treatment of Raji cells with flavonoids, quercetin resulted in effective inhibition of EA induction by TPA. None of the other flavonoids such as rutin, catechin and fi-naphthoflavone affected the induction of EBV-EA by TPA [59].

We reported that EBV replication induced oxidative stress in lymphoblastoid cell lines. In a previous study we have demonstrated that oxidative stress caused EBV lytic cycle induction as demonstrated by BZLF-1 gene expression. Thus, a vicious cycle may be initiated, whereby replication of EBV and ROS production amplify one another. This may lead to an increase in the number of EBV-infected cells and thus favor the development of EBV associated diseases, especially in immunodeficient individuals. The use of therapeutic drugs such as chemotherapy or immunosuppressive drug, which are known to induct oxidative stress may up-regulate the expression of BZLF-1, and may be a risk factor for EBV reactivation [60,61]. Inhibition of ROS may be useful in prevention or in the treatment of EBV-induced diseases.

In summary, EBV lytic cycle induction was able to damage lipids, proteins and DNA, crucial biomolecules in living cells. Same damages were observed in EBV associated malignancies such as DNA damage in nasopharyngeal carcinoma biopsies patients, and proteins modification in autoimmunes diseases. This investigation may provide important clues to the mechanisms involved in the development of cancer and autoimmunes deseases.

## Materials and methods

### Cell line and culture conditions

Cell lines and culture conditions

• Raji is a human Burkitt's lymphoma-derived cell line, harboring the latent form of EBV cycle [62].

• B95-8 is a lymphoblastoid cell line established from peripheral blood lymphocytes of a cotton-top marmoset (Saguinus Oedipus) following in vitro infection with EBV [37]. A small fraction (1-3%) of current batches of B95-8 cells spontaneously enter the viral lytic cycle.

All the cell lines were grown in RPMI 1640 medium (Gibco) supplemented with 10% fetal calf serum (FCS) and 2 mM L-glutamine in tissue culture flasks (Nunc). They were passaged twice a week and kept at 37°C in a humidified atmosphere of 95% air and 5% CO2.

### TPA treatment

For the induction of the lytic cycle, 3 × 10^6 ^cells were stimulated with 8 nM TPA for 2 h, when the cells were in logarithmic phase growth, usually 48 h after placing them in culture. The cells were washed two times with Phosphate buffer saline (PBS) and further incubated for 48 h in fresh culture medium [62].

### Preparation of cell extracts

Cells were centrifuged at 3,000 rpm for 10 min. The pellet was resuspended in 500 μl of deionized water and lysed by five cycles of sonication during 20 s at 37% (Sonisc, vibracell).

### Protein determination

Proteins were determined using the Protein Assay Kit from Bio-Rad (France) and bovine serum albumin served as the standard.

### Conjugated Dienes

Conjugated diene level was evaluated as described by Kurien and Scofield [63] with modification. 25 μl of cells lysat were extracted with 3 ml chloroform/methanol (2:1, v/v). After centrifugation at 3,000 rpm for 15 min, 2 ml of organic phase was transferred into another tube and dried at 45°C. The dried lipids were dissolved in 2 ml of methanol and absorbance at 233 nm was determined. It corresponds to the maximum absorbance of the extracted compounds.

### Determination of protein thiol levels

Protein thiols were quantified spectrophotometrically using 5,5-dithionitrobenzoic acid (DTNB); 250 ml of freshly prepared 10 mM DTNB in 0.05 M phosphate buffer pH 8, were added to 50 ml of cell lysat in 1200 ml of 0.05 M phosphate buffer. After incubation in the dark for 15 min at room temperature, the release of 5-thiobenzoic acid was quantified by measuring the absorbance at 412 nm and converted to absolute values using N-acetyl cysteine as standard (0-0.1 mM). A correlation coefficient of r2 = 0.999 was obtained. The absorbance of samples lacking DTNB was subtracted to account for the background absorbance at 412 nm. Samples were analysed in duplicate.

### Detection of MDA-protein

The MDA content was assessed by colorimetric assay, as described by Palacio et al [64]. This assay measures the MDA bound to proteins. In brief, cells lysats were precipitated twice with sulfuric acid and phosphotungstic acid. After centrifugation, the pellet was diluted with 800 ml of distilled water. Then, 10 ml of 0.14 mM ethylenediaminetetraacetic acid (EDTA), 80 ml of 0.2% butylated hydroxytoluene in ethanol (BHT) and 200 ml of 1% thiobarbituric acid (TBA) were added and incubated at 100°C for 60 min. An equal volume was extracted with butanol and centrifuged. Finally, 250 ml were removed from the butanolic phase and absorbances at 540 and 620 nm were determined with a Biochrom Libra S32 spectrophotometer. 1,1,3,3-Tetraethoxypropane (Sigma) was used as standard. A standard curve (0.05, 0.1, 0.25, 0.5, 1, 2.5, 5 nM) was included in every assay. A correlation coefficient of r2 = 0.989 was obtained. All the samples were treated in duplicate and the results are given as the mean values.

### Protein carbonyl (PC) levels

PC levels were measured according to the method based on spectrophotometric detection of the reaction of 2,4-dinitrophenylhydrazine (DNPH) with protein carbonyl to form protein hydrazones [30]. Briefly, after precipitation of protein with an equal volume of 1% trichloroacetic acid, the pellet was resuspended in 10 mM DNPH in 2 N HCl or with 2 N HCl as control blank. Next, after the washing procedure with 1:1 ethanol/ethylacetate, the final pellet was dissolved in 6 M guanidine. The carbonyl group was determined from the absorbance at 370 nm. The results were expressed as nanomoles of carbonyl groups per milligram of protein with an extinction coefficient of 22,000 M1 cm1.

### DNA fragmentation assay

After EBV lytic cycle induction, B958 and Raji cells were lysed in a buffer containing 10 mM Tris (pH 7.4), 150 mM NaCl, 5 mM EDTA and 0.5% Triton X-100 for 30 min on ice. Lysates were vortexed and cleared by centrifugation at 10 000 g for 20 min. Fragmented DNA in the supernatant was extracted with an equal volume of neutral phenol chloroform isoamyl alcohol mixture (25:24:1) and analyzed electrophoretically on 2% agarose gels containing 0.1 mg/ml of ethidium bromide [65].

### Statistical analysis

Statistical analysis was carried out by Student's t-test value, to assess the statistical significance of the obtained differences between treated and non-treated cells. A p < 0.05 was considered to be statistically significant.

## Abbreviations

EBV: Epstein Barr virus; TPA: 12- 0-tetradecanoylphorbol-13-acetate; TBA: thiobarbituric acid reactivity; MDA: malondialdehyde; SOD: superoxide dismutase; CAT: catalase; LPDs: lymphoproliferative diseases; PTLD: posttransplant lymphoproliferative disorder; SH: thiol; ROS: Reactive oxygen species; DNPH: 2,4-dinitrophenylhydrazine; CD: Conjugated dienes; OD: Optical density; TBA: Thiobarbituric acid; DTPA: Tris-cacodylic acid- diethylenetriaminepenta-acetic acid; H_2_O_2_: Hydrogen peroxide; PBS: phosphate buffer saline.

## Competing interests

The authors declare that they have no competing interests.

## Authors' contributions

BG and RN prepared the study design, carried out all the biological studies, analyzed and discussion of the data, and drafted the manuscript. MMJ helped with chemical analysis of the extract and correction of the manuscript. RBM carried out some biological assays and helped with the manuscript preparation. SL participated in the study design, discussion the data and helped to draft and correction of the manuscript. All authors have read and approved the final manuscript.
